# Advances in Laser Powder Bed Fusion of Tungsten, Tungsten Alloys, and Tungsten-Based Composites

**DOI:** 10.3390/mi15080966

**Published:** 2024-07-28

**Authors:** Hua Li, Yun Shen, Xuehua Wu, Dongsheng Wang, Youwen Yang

**Affiliations:** 1 School of Mechanical and Electrical Engineering, Jiangxi University of Science and Technology, Ganzhou 341000, China; 9120220032@jxust.edu.cn (H.L.);; 2 Key Laboratory of Construction Hydraulic Robots of Anhui Higher Education Institutes, Tongling University, Tongling 244061, China; 3 Institute of Additive Manufacturing, Jiangxi University of Science and Technology, Nanchang 330013, China

**Keywords:** additive manufacturing, tungsten, tungsten alloys, cracking, densification, tungsten alloys

## Abstract

In high-tech areas such as nuclear fusion, aerospace, and high-performance tools, tungsten and its alloys are indispensable due to their high melting point, low thermal expansion, and excellent mechanical properties. The rise of Additive Manufacturing (AM) technologies, particularly Laser Powder Bed Fusion (L-PBF), has enabled the precise and rapid production of complex tungsten parts. However, cracking and densification remain major challenges in printing tungsten samples, and considerable efforts have been made to study how various processing conditions (such as laser power, scanning strategy, hatch spacing, scan speed, and substrate preheating) affect print quality. In this review, we comprehensively discuss various critical processing parameters and the impact of oxygen content on the control of the additive manufacturing process and the quality of the final parts. Additionally, we introduce additive manufacturing-compatible W materials (pure W, W alloys, and W-based composites), summarize the differences in their mechanical properties, densification, and microstructure, and further provide a clear outlook for developing additive manufactured W materials.

## 1. Introduction

Tungsten, as a typical refractory metal, has a high melting point (3410 °C), high tensile strength (>900 MPa), high sputtering resistance, high hardness (>400 GPa), low thermal expansion coefficient (<4 μm·(m·k)^−1^), and excellent thermal conductivity (>150 W (m·k)^−1^), providing exceptional heat resistance and plasma radiation resistance. Its chemical properties are also very stable, with high resistance to arc erosion [1], and it exhibits high chemical stability against most molten metals and metal vapors. Due to its excellent properties, tungsten is widely used in military, medical, electronic, mining machinery, and nuclear fusion technology fields [1,2]. Applications include fusion plasma components [3], armor-piercing projectiles, kinetic energy penetrators [4], nuclear space propulsion engines, thermionic emitters in spacecraft [5], halogen lamp filaments, electronic emitters [6], armor-piercing projectiles, electrical switch contacts [7], rocket nozzles [8], collimators [9], wear-resistant parts for mining drills, nuclear radiation shielding materials, and protective components for X-ray and radiotherapy equipment.

However, tungsten belongs to the body-centered cubic (BCC) structure, which lacks closely packed planes and grain boundaries, resulting in relatively poor grain boundary cohesion [10]. Most thermal processes require high activation energy [11]. The dislocations within BCC metals are activated by the nucleation of kink pairs and the migration of screw dislocations due to the activation energy of thermal processes. The slip mechanism varies with temperature. At low temperatures, the slip system with high Peierls stress occurs because it has a sufficiently low activation energy barrier to generate kink pairs and enable dislocation movement. Generally, the internal dislocation migration rate is very low, limiting its plasticity. High resistance to dislocation movement leads to structural fracture before yielding. At high temperatures, the migration rate of screw dislocations gradually equals that of edge dislocations, and the phonon drag mechanism begins to control dislocation motion, causing plasticity before fracture, thus preventing cracking [10,12,13]. The results in a ductile-to-brittle transition temperature (DBTT) of tungsten typically ranging from 200 °C to 400 °C, with particularly low ductility at room temperature. The low recrystallization temperature leads to low plasticity and high susceptibility to cracking at room temperature [14]. The presence of lattice defects, impurities, and pores are the main factors leading to high DBTT in tungsten [15], and high DBTT is the primary cause of tungsten cracking, greatly limiting its manufacturability [16]. Additionally, during component manufacturing, the segregation of impurities such as oxygen, nitrogen, and carbon at the grain boundaries of tungsten (and its alloys) can cause intergranular fracture [17].

Therefore, tungsten is generally considered a brittle material, and cracking under external load is its primary mode failure [18]. This greatly limits the applicability of tungsten as a structural material. Due to the high brittleness of tungsten parts, mechanical processing is challenging. Currently, most tungsten parts are manufactured through powder metallurgy [19], powder injection molding [20], hot isostatic pressing, and thermomechanical work sintering [21]. Additionally, physical vapor deposition (PVD), chemical vapor deposition (CVD), and plasma spraying techniques are used to produce deposited tungsten films [22]. However, due to tungsten’s high hardness and brittleness at room temperature and its lack of ductility, these processing methods are not suitable for manufacturing complex structured components at room temperature. Furthermore, in the powder metallurgy process, the coarsening of ultrafine nanostructures can lead to heterogeneous microstructures during forming [23]. Therefore, traditional processing methods greatly limit the applications of tungsten materials.

In recent years, additive manufacturing (AM) has gained significant attention. Compared to traditional manufacturing technologies, it allows for selective layer-by-layer or particle-by-particle accumulation of materials by importing digital data models (CAD) files, and the entire workflow can be accomplished using various types of modeling software to complete part printing and post-processing. Additive manufacturing provides flexibility beyond traditional methods, allowing for the production of parts with complex external and internal structures without the need for preset molds, at lower processing costs. It enables cost reduction by modifying component shapes and achieving higher strength-to-weight ratios, thus reducing the overall weight of the manufactured parts [24,25,26]. Additionally, the high energy input and rapid cooling rates during the additive manufacturing process help inhibit phase transitions and the formation of metal compounds during shaping. Currently, additive manufacturing technology is widely used in the aerospace [27], automotive fields [28], biomedical [29], and defense industry [30].

In this review, to better understand the unique advantages and challenges of additive manufacturing methods for W materials, we will first introduce the basic principles of AMed W materials. Using physical equations and numerical phenomena, we will detail the multi-scale thermodynamic behavior of additively manufactured W and analyze its effects on the microstructural changes in grain growth. By discussing the impact of various additive manufacturing process parameters (including volumetric energy density, scanning strategy, substrate preheating, and oxygen content) on the performance of W manufacturing, we provide optimal process parameter references for future researchers. Subsequently, we will introduce representative W alloys and W-based composites to demonstrate the advantages of different materials in W manufacturing. This review offers researchers a comprehensive understanding of the field of additive manufacturing of tungsten materials and provides a solid foundation for high-quality AMed W materials.

## 2. Additive Manufacturing L-PBF

Compared to other additive manufacturing processes, L-PBF (Laser Powder Bed Fusion) is considered the most used AM technology for manufacturing W and W alloys. L-PBF is an additive manufacturing process initially proposed in the 1990s by German researchers Dr. M. Fockele and Dr. D. Schwarze of F & S Stereolithographie technik GmbH, along with Dr. W. Meiners, Dr. K. Wissenbach, and Dr. G [1]. This is a powder bed fusion process that uses high-intensity lasers as an energy source to selectively melt and fuse regions of metal powder layer by layer by importing 3D volumetric data models (CAD files). Additive manufacturing technologies applied to metals are mainly divided into Direct Energy Deposition (DED) and Powder Bed Fusion (PBF), which can be distinguished by their different heat sources (laser, electron beam, plasma arc, etc.). Among them, Powder Bed Fusion technology distributes metal powder layer by layer on a powder bed and uses high-energy beams (such as laser or electron beam) as an energy source to process refractory metals such as W [31], Ta [32], Mo [33], and Zn [34]. During the manufacturing process, the high-energy beam melts and solidifies the selected areas of each layer according to a preset path, building the part layer by layer [1]. In laser beam-based PBF, the Selective Laser Melting (SLM) process is a widely used, advanced additive manufacturing process that uses high-energy lasers to melt metal powders [35], allowing precise control over the melting process, minimizing material waste, and optimizing structural performance. It produces parts with excellent mechanical properties, smooth surfaces, and complex structures [36,37].

Due to the mechanical properties and lightweight characteristics of the L-PBF process, it is widely used in aerospace, electronics, automotive, and biomedical fields [38,39]. In the automotive industry, L-PBF technology uses various materials to manufacture lightweight structural components, functionally complex parts, and high-performance engine components, achieving optimized part design, such as integrating multiple parts into a single complex component, thereby reducing assembly requirements and improving production efficiency, while also optimizing part weight [39]. In the medical field, L-PBF technology can customize the production of implants based on patient body conditions using various materials (such as magnesium alloys, titanium alloys), including hip joints, spine, and dental implants. Additionally, by optimizing design and altering microstructures to promote bone tissue growth, it improves biocompatibility and implant success rates [38]. In L-PBF, residual stress and shrinkage are phenomena that need attention during the manufacturing process. The thermal effects of high-density laser energy cause material expansion during processing, followed by shrinkage after the laser is removed. Additionally, the setting of process parameters also affects the internal residual stress of the material. This can lead to the production of defective parts [40]. This review will detail the printability of pure tungsten, tungsten alloys, and tungsten-based composites using L-PBF.

### 2.1. Multiscale Thermodynamic Behavior of W in L-PBF Additive Manufacturing

**Figure 1:**
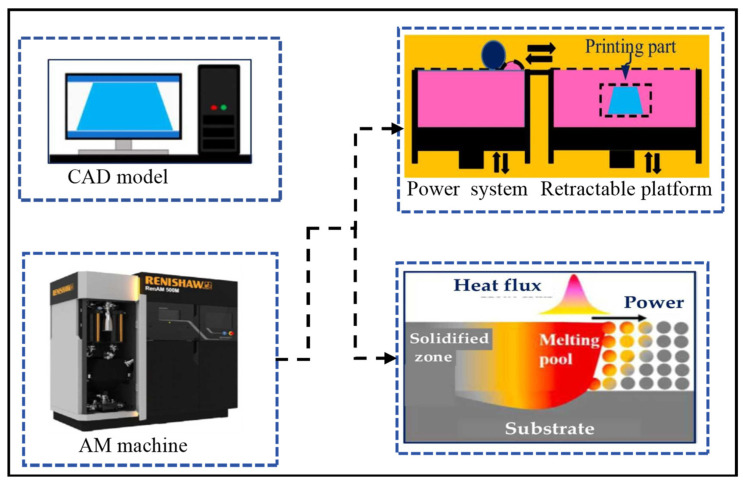
Schematic diagram of the L-PBF system working principle [43,44,45,46].

Laser Powder Bed Fusion L-PBF can be divided into Selective Laser Melting (SLM) and Selective Laser Sintering (SLS). For tungsten with a very high melting point, SLM and SLS can occur simultaneously within the same framework in L-PBF [41]. The Laser Powder Bed Fusion (L-PBF) process is typically conducted in an enclosed inert gas environment to reduce the impact of oxygen content during the manufacturing of parts. As shown in Figure 1, it consists of a vertically movable platform, powder delivery system, roller, laser system, and base plate. Initially, by selecting different scanning strategies, the laser beam melts specific areas of the uniformly deposited powder bed, where the powder particles absorb energy and melt to form a melt pool, constructing the first layer of the part. After the first layer is complete, the platform is lowered to reduce the layer thickness, and a new layer of powder is deposited with the roller, repeating this process to achieve the printing of tungsten parts [42].

To understand the manufacturing process of W in additive manufacturing, we will first explain the general behavior and characteristics of W‘s melt pool. The heat input from the laser source to the material in the powder bed causes local melting of the powder bed and the formation of a melt pool. The morphology and thermodynamic distribution of the melt pool directly affect the melting and solidification of W material, impacting the final microstructure and mechanical properties formed. To accurately describe the plastic deformation and residual stresses generated in the additive manufacturing process, and to further study the mechanisms by which these affect the microstructure, a sequentially coupled thermomechanical model is typically used to calculate the changes in temperature and stress during the AM process [47].

#### 2.1.1. Thermodynamic Analysis

Rosenthal [48] first applied Fourier’s law to moving heat sources, representing them as point, line, or surface sources. While this provided reasonable predictions for transient temperature fields at some distance from the heat source, it was not very accurate near the fusion zone. Many researchers [49,50] assume that thermal energy diffuses outward from the center of the laser beam in a Gaussian distribution. This model, due to its simplicity, is widely used in laser processing, although it is not sufficiently accurate for simulating thermal accumulation and multiple thermal cycles (multi-layer multi-pass welding). Goldak et al. [51] proposed a three-dimensional asymmetric heat source based on a double-ellipsoidal heat power density distribution, which better accounts for depressions on the melt pool surface caused by arc pressure. The non-equilibrium nature of the temperature curves considered in Goldak’s double-ellipsoidal heat source model accurately reflects the thermal processes in AM and has been proven very effective in various laser and electron beam welding techniques. The expression for its heat conduction equation is as follows:

(1) $$\Delta \cdot \left(k\Delta T\right)+Q=\rho C_{p}\frac{\partial T}{\partial t}$$

where *T* is the temperature, Δ⋅(kΔT) is the heat diffusion, *Q* is the heat source strength per unit volume, k is the thermal conductivity, ρ is the material density, Cp is the specific heat capacity, ∂T/∂t is the rate of change temperature over time. The heat source *Q* can be expressed in the following form:

(2) $$Q\left(x,y,z\right)=\frac{6\sqrt{3}Q_{f}}{\pi abc}\exp\left(-3\left(\frac{{\left(x-x_{0}\right)}^{2}}{a^{2}}+\frac{{\left(y-y_{0}\right)}^{2}}{b^{2}}+\frac{{\left(z-z_{0}\right)}^{2}}{c^{2}}\right)\right)$$

where $Q_{f}$ is the total heat input during the welding process. *α*, *b*, and *c* are the semi-axes lengths of the ellipsoid in three directions, ($x_{0}$, $y_{0}$, $z_{0}$) is the position of the heat source center.

In the melt pool, the flow behavior of the molten metal inside the pool is controlled by its dynamic viscosity, which in turn affects the distribution of thermal energy within the pool and the stability of the pool. The dynamic viscosity of molten W (*λ*) is defined as follows [52]:

(3) $$\lambda =\frac{15}{16}\mu \sqrt{\frac{m}{KT}}$$

where *m* is the atomic mass, *K* is the Boltzmann constant, *T* is the temperature of the melt pool, μ is the surface tension of the melt pool. From this, it is evident that the surface tension of W has an inverse linear relationship with temperature; as the temperature increases, the surface tension of the melt pool decreases, thus reducing the dynamic viscosity of molten W, enhancing the fluidity of the melt pool, and increasing the density of recirculation, which helps reduce the formation of keyhole depressions. On the other hand, the melt pool is influenced by Marangoni effect dynamics, causing the melt pool to flow inward and outward, depending on the local temperature. With the increase in laser input energy, the Marangoni flow also increases, which enhances the probability of gas dragging into the melt pool, thereby increasing the porosity within the melt pool [53]. The surface of the melt pool is also influenced by the interface force of the recoil pressure, which acts on the free surface and pushes the liquid downwards, and in certain cases, the recoil pressure can lead to the formation of keyhole depressions, thus increasing the porosity of the melt pool [54]. Simultaneously, by alloying W material with other elements (Ni [55], Ta [56], Nb [57], Cu [58], etc.), the average effective atomic mass is reduced, thereby significantly lowering the dynamic viscosity and enhancing the quality of additive manufacturing of W. As theoretical, modeling, and experimental studies continue to deepen, the development of melt pool dynamics will become more detailed; through these descriptions, along with the thermophysical properties related to W materials, a detailed model of melt pool boundaries and flow dynamics can be established.

#### 2.1.2. Crystal Plasticity Analysis

Tungsten is a typical body-centered cubic (BCC) material, lacking closely packed planes and grain boundaries [10]. Screw dislocations are caused by the thermal activation of kink pair nucleation and the migration of spiral dislocations [11]. Better reveal the mechanisms of microstructural evolution during the additive manufacturing of tungsten, a crystal plasticity model of tungsten is established in thermodynamics to analyze the effects of motion induced, plastic, dislocation strain on grain size and residual stress. The multiplicative decomposition of the deformation gradient in the single crystal plasticity model is expressed as follows [59]:

(4) $$E=E_{e}\cdot E_{t}\cdot E_{P}$$

where E is the total deformation gradient, $E_{e}$ is the elastic part, $E_{t}$ is the thermal deformation part, and $E_{P}$ is the plastic part. Deformations caused by solid-phase changes and creeps are relatively minor and can be neglected. The total velocity gradient (*V*) is defined as follows [60]:

(5) $$\begin{array}{cc} V & ={\overset{\cdot }{E}}_{e}E_{e}^{-1}+E_{e}{\overset{\cdot }{E}}_{t}E_{e}^{-1}E_{t}^{-1}+E_{e}E_{t}{\overset{\cdot }{E}}_{p}E_{e}^{-1}E_{t}^{-1}E_{p}^{-1}\\ & =V_{e}+V_{t}+V_{p} \end{array}$$

where $V_{e}$ is the elastic velocity gradient, $V_{t}$ is the thermal velocity gradient, $V_{p}$ is the plastic velocity gradient and $\dot{E}$ is the time derivative. This can be considered as an additive decomposition of the velocity gradients. The thermal deformation gradient $V_{t}$ and its time derivative can be calculated as follows:

(6) $$E_{t}=\lambda \left(T\right)\sigma$$

(7) $${\overset{\cdot }{E}}_{t}=\frac{\partial \lambda }{\partial T}\overset{\cdot }{T}\sigma$$

(8) $$V_{t}=E_{e}\overset{\cdot }{E_{t}E_{e}^{-1}E_{t}^{-1}}=\frac{1}{\lambda \left(T\right)}\sigma \frac{\partial \lambda }{\partial T}\overset{\cdot }{T}=\alpha_{T}\overset{\cdot }{T}\sigma$$

where *λ*(*T*) is the thermal strain rate, $\alpha_{T}$ is the thermal expansion coefficient, and *σ* is the second-order identity tensor. The plastic velocity gradient $V_{p}$ can be expressed as follows [50]:

(9) $$V_{p}=E_{e}E_{t}{\overset{\cdot }{E}}_{p}E_{e}^{-1}E_{t}^{-1}E_{p}^{-1}=E_{e}{\hat{V}}_{p}E_{e}^{-1}$$

where ${\hat{V}}_{p}$ is a key variable, the plastic velocity gradient in the intermediate configuration, related to the plastic mechanism, and can be calculated as follows [61]:

(10) $${\hat{V}}_{p}=\sum \beta \overset{\cdot }{\mu^{\beta }}m^{\beta }n^{\beta }$$

where the superscript *β* denotes variables corresponding to the *β*th slip system, $\dot{\mu^{\beta }}$ is the slip rate, and the unit vectors $m^{\beta }$, $n^{\beta }$ represent the slip direction and the normal vector to the slip plane.

#### 2.1.3. Evolution of Microstructure

In additive manufacturing of tungsten, the formation of thermal strains and residual stresses during the melting and solidification process initiated by the high-energy laser beam on metal W particles is complex and significant. Once the high-energy laser beam leaves the melt pool area, the metal material rapidly cools and begins to solidify. During the very short time the material is near its melting point, its yield stress is very low. Due to the expansion of the preceding melt pool, the metal material yields under compression. As the laser moves forward, the material behind the melt pool continues to cool, undergoes tensile stress, and yields again, ultimately resulting in the solidified tracks being under tensile stress while the adjacent built tracks are under compressive stress. Rapid cooling leads to grain refinement and lattice distortions, thereby creating significant residual stresses inside the material [62]. The manifestation of these residual stresses is as follows [63]:

(11) $$\sigma =\frac{E}{\left(1+\nu \right)\left(1-2\nu \right)}\left(\nu \partial_{ij}\varepsilon_{kk}+\left(1-2\nu \right)\varepsilon_{jj}-\left(1+\nu \right)\alpha \Delta T\partial_{ij}\right)\left(i,j,k=1,2,3\right)$$

where *E* is the elastic modulus of the material (W is 350–410 GPa), *v* is the Poisson’s ratio (W is 0.28–0.3) [1]. $\partial_{ij}$ is the Kronecker delta, i≠j is 0, i=j is 1, α is the thermal expansion coefficient of the material, is the total strain from mechanical and thermal strains, and ΔT is the temperature change. According to Bragg’s equation [64]:

(12) 2dsinθ=nλ

where *d* is the interplanar spacing, λ is the wavelength of X-rays, θ is the diffraction angle, and n is the order of diffraction. According to Equation (12), is smaller, is larger, and the greater the residual stress and lattice expansion in W produced by additive manufacturing.

These residual stresses are particularly important when cutting samples and separating them from the base plate, as they may cause unnecessary deformation and deflection, thereby affecting the final quality and precision of the parts. These stresses can be mitigated to some extent by optimizing process parameters (such as laser power and scanning speed), preheating the substrate, and appropriate post-processing (such as heat treatment) to improve the overall performance of the parts [65].

For traditional processing techniques such as sintering, the manufacturing process of W involves powder densification and grain growth. Research by Du, Z. et al. [66] shows that the densification rate of W first increases and then decreases with rising temperature, reaching a distinct peak at about 1450 °C. The process of grain growth is divided into four stages: particle contact, decreasing orientation angles of grain boundaries, disappearance of grain boundaries, and grain growth. Initially, there are pores between the grains; as the sintering temperature rises, the grains grow and form clear boundaries, the pores between grains essentially disappear, and at high temperatures, the energy of the grain boundaries tends to decrease as their orientations become aligned and the orientation angles diminish. When the orientations of the grain boundaries become uniform, the boundaries gradually disappear, leading to an increase in grain size. When the temperature is below 1700 °C, atoms move to grain boundaries by diffusion, forming distinct necks in the powder, the porosity gradually decreases, but the growth of grain size is relatively slow. When the temperature ranges from 1700 °C to 2000 °C, the growth rate of tungsten grains significantly increases, achieving a maximum relative density of 96.9% with an average grain size of about 5 um. This indicates that the dynamics and kinetics of densification and grain growth during traditional tungsten processing are completely different; densification processes are more pronounced in the early and mid-stages of processing, while grain growth processes are more significant in the later stages.

In the additive manufacturing process, the treatment of tungsten differs drastically from traditional powder processing techniques. In additive manufacturing, tungsten first melts in the melt pool, then undergoes nucleation and solidification, ultimately forming grains. The heat treatment in additive manufacturing is contrary to traditional processing methods, meaning that as the temperature decreases from a specific melting point, it skips the traditional densification process. Moreover, compared to traditional near-equilibrium processing methods such as sintering, the melting and solidification processes in additive manufacturing can be repeated, such as through multiple remelting processes, offering a way to process materials in a rapidly changing thermal environment. This method allows for multiple remelting and solidification of materials in a very short period, providing greater operational flexibility and control compared to traditional sintering processes. However, in both manufacturing technologies, grain growth is influenced by diffusion, grain boundary migration, and recrystallization, with increasing temperature also promoting grain growth and recrystallization. The relationship between grain size and temperature during the sintering process can be expressed as follows [67,68]:

(13) $$G_{t}^{n}-G_{0}^{n}=k_{0}t\exp\left(-\frac{Q_{G}}{RT}\right)$$

where $G_{t}^{n}$ and $G_{0}^{n},$ respectively, represent the grain size at *t* = *t* and the original grain size at *t* = 0, the exponent n depends on the mechanism controlling grain growth, with *n* = 1 indicating the surface diffusion mechanism; *n* = 2 indicating the volume diffusion mechanism; *n* = 3 indicating the lattice diffusion mechanism; *n* = 4 indicating the grain boundary diffusion mechanism. $k_{0}$ is a constant related to the material and kinetics, $Q_{G}$ is the activation energy of grain growth, R is the gas constant, and T is the processing temperature.

For non-isothermal sintering processes (such as additive manufacturing), Wang [68] proposed another form to express grain growth, where the relationship between temperature and grain size can be expressed as follows:

(14) $$\ln\left(\frac{G_{t}^{n}-G_{0}^{n}}{T}\right)=\ln\left(\frac{Rk_{0}}{Q_{G}}\right)-\frac{Q_{G}}{R}\times \frac{1}{T}$$

where β is a constant heating rate dT/dt, and other symbols follow Equation (14). The values of n and $Q_{G}$ can be determined by plotting $In(G_{t}^{n}-G_{0}^{n}/T)\;vs.\;(1/T)$, where *n* and $Q_{G}$ are derived from the best linear regression coefficients and the slope of the linear relationship. In additive manufacturing, grain growth in tungsten is typically governed by surface diffusion, denoted as *n* = 1. The rapid cooling rate in AM, *T* is considered a short-term peak in melting temperature.

By comparing the melting and grain growth mechanisms of tungsten (W) in traditional processing and additive manufacturing, the dynamic characteristics of W under different processing techniques are revealed. This comparison allows for better prediction of the behavior of tungsten during manufacturing, providing a theoretical foundation and practical guidance for optimizing and developing tungsten-based processing techniques, thus facilitating the application of high-performance tungsten in advanced manufacturing.

## 3. Additive Manufacturing of Pure Tungsten via L-PBF

Compared to traditional processing techniques, the additive manufacturing L-PBF technology, with its high energy input and rapid cooling rates, can inhibit phase transformations and the formation of intermetallic compounds, offering unique advantages in the fabrication of tungsten components [69,70]. Enneti et al. [71] utilized non-spherical and irregular-shaped W powder to study the effects of hatch spacing and laser scanning speed on the densification of W parts, achieving a maximum relative density of 75%. Wang et al. [72] used spherical tungsten powder to examine how scanning strategies affect crack distribution in tungsten samples processed by LPBF, and achieved a higher density (96.5%) in bulk tungsten by optimizing the scanning strategies. Zhou et al. [73] successfully fabricated two types of porous tungsten skeletal structures, honeycomb and square frameworks, using L-PBF technology. The results indicate that these two skeletal structures have porosities of 52 vol% and 68 vol%, compressive strengths of 256 MPa and 149 MPa, respectively, with the grains showing equiaxed morphology on the XY plane and columnar morphology on the YZ/XZ planes (parallel to the *Z*-axis), displaying anisotropy with the highest values along the *Z*-axis. The fracture morphology along the *Z*-axis exhibits trans-granular cracking and tearing characteristics, while along the *X*-axis, the cracks show only intergranular fracture characteristics.

**Figure 2:**
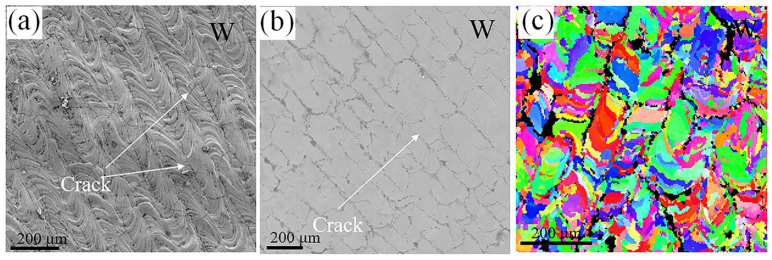
The crack distribution on the top surface of pure tungsten. (**a**) the raw surface morphology, (**b**) the crack distribution and (**c**) the EBSD of pure W [75].

Currently, most scholars find that tungsten parts manufactured using the L-PBF process exhibit certain cracks, which are believed to be caused by the combined effects of thermal stresses from uneven heating and cooling during the manufacturing process and the behavior of the material below its DBTT [23]. If internal thermal stresses exceed the ultimate tensile strength, cracks will occur. Typically, in L-PBF manufacturing of tungsten, longitudinal cracks parallel to the laser scanning direction and transverse cracks inclined to the laser scanning direction can be observed. Vrancken et al. [74] analyzed the microcrack formation mechanisms in tungsten during the L-PBF process through numerical simulations and in situ experiments. The study shows that microcracks form within a narrow temperature range and are related to strain rates. The largest Von Mises residual stress affects the size of the crack zones around the scanning tracks, with the crack morphology primarily depending on the local direction of the principal stress. Wang et al. [75] manufactured pure W parts using L-PBF, as shown in Figure 2. The surface morphology and microstructure of pure W, indicated by white arrows in the top surface EBSD images, clearly show numerous long and straight cracks, with a measured surface crack density of 10.1 mm^−1^. Farid et al. [18] studied the cracking behavior of pure tungsten under long laser pulse loads. In the SLM manufacturing process of tungsten, laser parameters, scanning strategies, substrate preheating, and hatch spacing significantly affect the final microstructure. For tungsten, the maximum durable temperature difference is 800 K, according to the cracking criteria proposed in the literature [72]. Under L-PBF laser melting, the calculated temperature difference is 7300 K, far exceeding the critical temperature gradient. Therefore, cracking in tungsten parts manufactured by L-PBF is inevitable. Although pure tungsten has relatively poor ductility, the thermal stresses generated during the L-PBF manufacturing process can be mitigated by adjusting laser power, substrate preheating, and other process parameters [76,77]. This article explores changes in the following process methods:

### 3.1. Process Parameters

The L-PBF process involves numerous physical phenomena such as the absorption and reflection of laser radiation, phase changes, fluid flow, vaporization, heat transfer, chemical reactions, and convection. Simultaneously, during the powder melting process, phase changes from solid to liquid and liquid to solid occur simultaneously [78]. Understanding the physical phenomena in the L-PBF manufacturing process and optimizing the processing parameters helps in producing defect-free parts. By modifying specific parameters based on material characteristics, it is possible to print components with low residual stress, low deformation, and excellent quality [79].

#### 3.1.1. Volumetric Energy Density

Volumetric Energy Density (VED) is a key characteristic in the manufacturing of molten materials, representing the energy transferred per unit volume of powder material deposited in the powder bed layer [80]. According to the definition of the VED formula proposed by Ciurana et al. [81], VED is influenced by four independent process parameters: laser power *P* (energy intensity of the laser beam), scanning speed *v* (speed of the laser beam movement), powder bed layer thickness t (layer thickness equal to the increment of the powder bed), and hatch distance (distance between two adjacent lines). Ciurana’s empirical formula for *VED* is calculated using Equation (15):

(15) $$VED=\frac{P}{vt\sigma }$$

The laser absorption rate and thermal properties of different materials vary, and the value of volumetric energy density must be established through extensive experimental work. Relatively speaking, higher energy density values lead to deeper and wider melt pools [82]. Haijun et al. [83,84] found a correlation between VED values and the porosity within parts manufactured using the SLM process for Ti-6Al-4V alloy. When the energy density delivered to the powder bed is insufficient, a significant amount of porosity exists within the manufactured parts, adversely affecting the tensile strength and fatigue strength of the parts. It was also found that reducing the layer thickness of the printed parts can reduce residual stress and deformation in the material. In the SLM manufacturing process, lower laser power results in shallower melt depths, reducing energy transfer and hindering metallurgical bonding with the previous layer. Higher laser power leads to increased melt depths, creating high thermal stresses and causing surface cracks. Therefore, selecting the appropriate laser power facilitates powder melting and melt flow, resulting in small-angle grain boundaries between scan tracks [23,85].

The amount of energy input determines the heat transfer modes in the L-PBF manufacturing process: conduction mode and keyhole mode. At high energy densities, the material within the melt pool becomes turbulent due to intense vapor pressure, resulting in larger pores and defects in the solidified parts. Printing in conduction mode is more likely to achieve stable melt tracks with fewer defects [78]. Yang et al. [86] studied the threshold of the keyhole mode, showing that the most critical factor affecting energy density is scanning speed. At lower scanning speeds, the energy input interacts with the powder for a longer duration, potentially promoting the keyhole mode. Vapor recoil force causes melt track deformation, leading to a poor powder deposition and lower relative density of the printed parts. Chen et al. [87] measured the mechanical properties of W70 composites fabricated by Laser Powder Bed Fusion (L-PBF) at different energy densities. The results showed that when the energy density was 300 J/mm^3^, the ultimate tensile strength (UTS) was only 950 MPa. However, when the energy density increased to 390 J/mm^3^, the UTS reached 1060 MPa. This indicates that the tensile strength of W70 composites significantly improves with increasing energy density. Nevertheless, excessive energy input may lead to void formation due to melt pool instability. Yuan et al. [88] investigated the effects of laser scanning speed on the fabrication of an SS316L material using the SLM process. The results indicate that scanning speed significantly alters melt pool distribution, residual stress, and grain distribution. Extremely low scanning speeds result in excess porosity within the melt pool, leading to a decrease in volumetric density. Therefore, selecting an appropriate scanning speed is crucial for managing the scan tracks in L-PBF manufacturing of tungsten.

**Figure 3:**
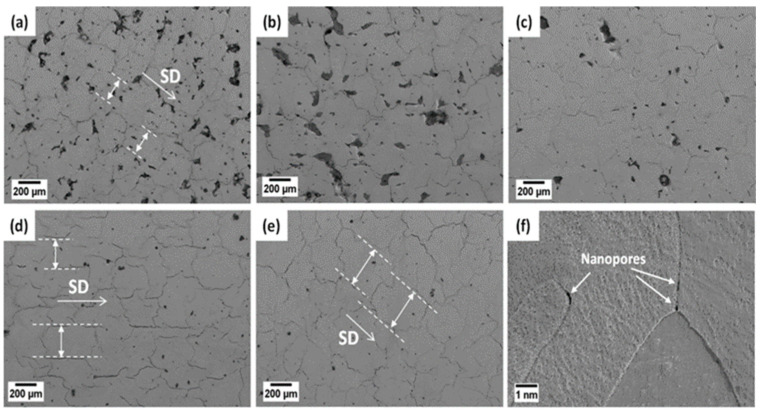
SEM micrographs of the top view of pure W samples prepared by L-PBF: (**a**–**f**) nanopores formed in the as-built samples [89].

Shi [89] studied the effects of different VEDs on the microstructure of pure W samples prepared by L-PBF. As shown in Figure 3a, numerous irregular pores and cracks with sizes ranging from 10 to 200 μm were observed in samples with a VED of 156.25 J/mm^3^, with the distance between two independent longitudinal cracks measured to be approximately 190 μm. With the increase in VED, the number and size of these pores gradually decreased, and the distance between cracks increased, as shown in Figure 3b–e. When the VED increased to 364.58 J/mm^3^, the large pores essentially disappeared, with the remaining small pores having a diameter of about 5–20 μm, and the distance between cracks was 360 μm. This is because, as the VED increases, the temperature of the melt pool rises, reducing the viscosity of the fluid, thereby enhancing the wetting and spreading of the molten droplets. Additionally, in Figure 3f, some tiny nano-pores were observed to be distributed along the grain boundaries.

Hatch spacing and layer thickness are also important factors affecting volumetric energy density. During the laser scanning process, repeatedly remelting parts with overlapping hatches is expected to eliminate defects. The thicker the layer, the more energy is required for the laser to melt the thick powder bed. Mishra et al. [90] found a linear relationship between hatch spacing, layer thickness, and the overlapping cross-section of the melt pool. Based on Equation (15), as hatch spacing and layer thickness increase, the volumetric energy density input decreases. Therefore, using a smaller powder bed thickness will produce conduction mode tracks. Enneti et al. [71] studied the effects of process parameters on tungsten materials using L-PBF manufacturing, showing that parts produced at lower scanning speeds and hatch spacing have higher density. Yang et al. [91] investigated the impact of hatch spacing on the microstructure, phase, and nano-hardness of TiAl/TiB processed by L-PBF. The results show that as hatch spacing increases, remelting time shortens, leading to a decrease in the extent of recrystallization. Consequently, the volume fraction of HAGBs decreases with increasing hatch spacing.

#### 3.1.2. Scanning Strategy

**Figure 4:**
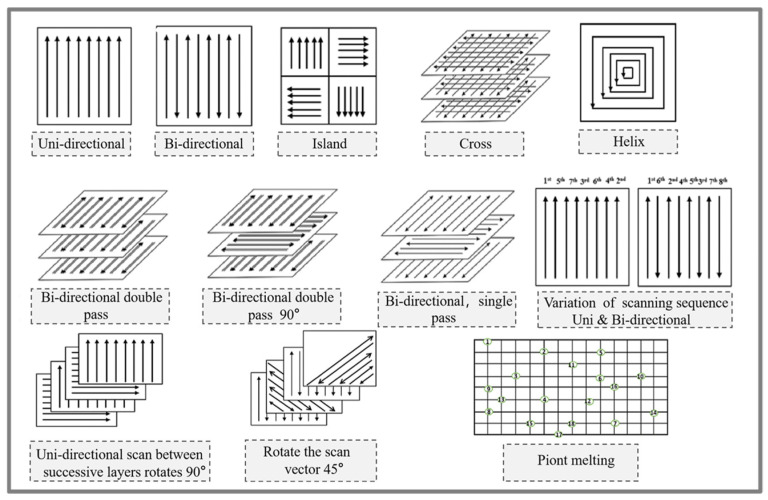
Common types of scanning strategies [100,101].

In the L-PBF printing process, different laser scanning strategies can affect the residual stress, grain boundary structure, and grain morphology within the printed parts, leading to varying degrees of densification and changes in microstructure and texture, resulting in different mechanical properties of the produced samples [92]. Early scholars believed that the greatest residual stress occurs perpendicular to the scanning direction [93,94], while other studies indicate that the greatest residual stress is parallel to the scanning direction [95,96]. Robinson et al. [97] suggested that using shorter scan vector lengths can minimize residual stress in printed parts. Therefore, selecting an appropriate laser scanning strategy helps to produce clear textures or more uniform crystal orientations [98]. Geiger et al. [99] demonstrated that different laser scanning strategies can affect the texture of IN738LC processed by L-PBF, thereby influencing the anisotropy of elastic properties. Figure 4 illustrates various scanning strategies used to alter the density and texture of printed parts. Among them, unidirectional, bidirectional alternate, and island (chessboard) scanning strategies are the most commonly used in L-PBF. Using a unidirectional scanning strategy with layer-by-layer alignment, the movement direction of the energy beam remains the same, usually producing an anisotropic stress state, leading to insufficient densification and strong textures [100]. Bidirectional alternate scanning, also known as zigzag scanning, differs from the unidirectional strategy as the energy beam’s movement direction is opposite on adjacent parallel scan vectors [87,101]. The island (chessboard) scanning strategy divides the area into small square units, forming a chessboard pattern that reduces scan vector length [102]. Spiral scanning strategies can reduce the length of scan vectors, thereby decreasing the accumulated stress from overly long scan lines [103]. Double pass laser scanning, as one of the laser rescanning strategies, can typically refine the microstructure of printed materials [104]. The single-point melting scanning strategy differs from other continuous scans, selecting one point at a time with variable parameters of laser beam dwell time and point spacing [100].

**Figure 5:**
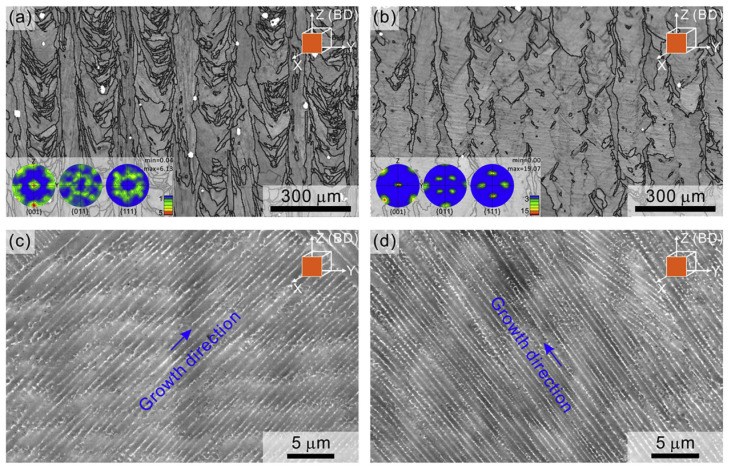
Band contrast (BC) maps showing grain structure of the (**a**) SS-X and (**b**) SS-XY specimens from the YZ cross section, insets in (**a**,**b**): pole figures of the SS-X and SS-XY specimens, respectively, high-magnification SEM backscatter electron images of the dendrite structure of the (**c**) SS-X and (**d**) SS-XY specimens [106].

Luke et al. [105] used the island scanning strategy, which strongly influenced the grain structure and internal residual stress of printed parts, thereby reducing the cracking behavior of the parts. J. Robinson et al. [97] showed that unidirectional scan vectors generate high stress in the parallel direction and low stress in the perpendicular direction. The bidirectional alternate scanning strategy provides the most uniform distribution and measures the lowest residual stress among the multidirectional scanning strategies. Wan et al. [106] used the L-PBF process to explore the effects of bidirectional scanning without interlayer rotation and bidirectional scanning with continuous interlayer rotation of 90° on the mechanical properties of INC718 material. The results show that samples with bidirectional scanning without interlayer rotation exhibit weak cubic texture, while samples with continuous interlayer rotation of 90° bidirectional scanning exhibit strong cubic texture. Additionally, samples with bidirectional scanning without interlayer rotation have higher tensile strength and fatigue strength, as shown in Figure 5.

Thijs et al. [107] altered scanning strategies in the SLM process to study the microstructural evolution of Ti-6Al-4V, finding that local heat transfer conditions associated with the scanning strategies can affect the growth direction of elongated grains. In the unidirectional scanning strategy, elongated grains grow towards the melt pool and are parallel to each other. In the bidirectional scanning strategy with 90° rotation between consecutive layers, the grains become more equiaxed, forming a grid-like planar structure. Geiger et al. [99] used three different scanning strategies: A: Scan vectors rotate 90° (parallel/perpendicular to the reference direction), B: Scan vectors rotate 90° (+45°/−45° to the reference direction), C: Scan vectors rotate 67° (pseudo-uniform around the building direction). The results showed that scanning strategies A and B developed cubic textures, while samples built with scanning strategy C exhibited fibrous textures with a fairly even distribution of <100> and <110> on the scanning plane. The grains in scanning strategy A were smaller than those in scanning strategy B, and the strength of the crystal texture was significantly enhanced. Larimian et al. [108] found that interlayer rotation can reduce porosity and form a more uniform structure by remelting deposited layers in different directions. Variations in the scanning rotation angle between layers in additive manufacturing can significantly impact the strength and ductility of the built parts [109]. Therefore, it is best to use scanning strategies with interlayer rotation to reduce the texture and anisotropy of the built samples.

#### 3.1.3. Substrate Preheating

Substrate preheating is a strategy to improve the material properties of parts printed by the L-PBF process. On the one hand, it can reduce the temperature gradient, thereby decreasing the residual stress generated during the L-PBF manufacturing process. On the other hand, it can avoid the DBTT of tungsten, which ranges from 200 °C to 400 °C. Printing tungsten parts at room temperature may lead to brittleness during the solidification and cooling stages. Theoretically, if the substrate preheating temperature is higher than the DBTT, the screw dislocations in tungsten will have sufficient mobility to alleviate plastic deformation caused by the temperature gradient during solidification and cooling [43,74,110]. Kevin et al. [111] studied the effect of preheating conditions on deformation and residual stress distribution using a finite element model. The results showed that under conditions of no preheating, single, and multiple preheating, the initial layer exhibited the maximum tensile stress. As the number of deposited layers increased, the final deposited layer exhibited the highest compressive stress, with the peak tensile stress gradually decreasing with the number of preheating cycles. Experiments showed that under no preheating conditions, the longitudinal residual stress increased layer by layer from the bottom to the top of the substrate. With the addition of preheating, the stress distribution curve in the substrate changed, and a noticeable stress minimum appeared with increasing preheating cycles. This is because the additional energy introduced by preheating effectively reduced the temperature gradient within the build plate, helping to decrease the thermal expansion mismatch between the substrate and the deposited layers, thereby reducing stress concentration caused by inconsistent thermal expansion and significantly lowering the residual stress.

A.v. Müller et al. [43] increased the substrate preheating temperature to 1000 °C when manufacturing pure tungsten using the SLM process. The results showed that compared to preheating temperatures of 600 °C and 800 °C, increasing the substrate preheating temperature had a positive impact on alleviating cracks in the parts, achieving high densification (98% relative density), but it could not completely eliminate the formation of crack defects. Literature [56] also indicated that for pure tungsten with a grain size of 10–100 μm, preheating the substrate to 400 °C was insufficient to mitigate the thermal stress during solidification, and cracks were not significantly improved. Preheating the substrate to a certain temperature in advance can alleviate the formation of cracks, but it cannot completely eliminate them. In the study by Vrancken B et al. [112], the issue of microcracks in W was not considered an insurmountable problem. They found that increasing the preheating temperature gradually reduced the occurrence of cracks, and preheating above 773K–873K completely eliminated the cracks. They suggested that, compared to other studies, the ability to fabricate crack-free W using L-PBF was attributed to the low oxygen content in their W powder, which requires further investigation. Manufacturing defect-free tungsten through L-PBF remains a significantly challenging task.

### 3.2. Impact of Oxygen Content

The level of oxygen content is crucial in the additive manufacturing process of W. Typically, L-PBF manufacturing of W is conducted in a vacuum environment, but it can still be affected by small amounts of oxygen, which may come from residual oxygen in the chamber or from the oxidized powder surface. Ivekovic et al. [56] used L-PBF technology to manufacture tungsten parts and raised the substrate temperature to 400 °C, which is above the inherent DBTT of tungsten. The research found that the presence of oxide impurities at the grain boundaries increased the DBTT to above 400 °C. Further comparing different sealing equipment, they found that the increased oxygen content in the building chamber led to the separation of oxygen at the grain boundaries as tungsten oxide during the rapid solidification process in AM, weakening the grain boundaries and causing thermal cracks. When the oxygen content in the chamber was 200 ppmv, compared to equipment with less than 50 ppmv, the density of the manufactured parts decreased from 97.1% to 94.4%. Enneti et al. [71] conducted processing of tungsten powder under normal conditions, leading to a higher oxygen content in the powder. The maximum relative density of the manufactured tungsten parts was only 75.7%, whereas other researchers achieved relative densities of 96–98% using plasma-spheroidized powder under the same parameters [52,72]. As shown in Table 1, the summary statistics of the effect of different oxygen content of the building chamber on the structural densities and microstructures of the tungsten materials prepared in L-PBF.

**Figure 6:**
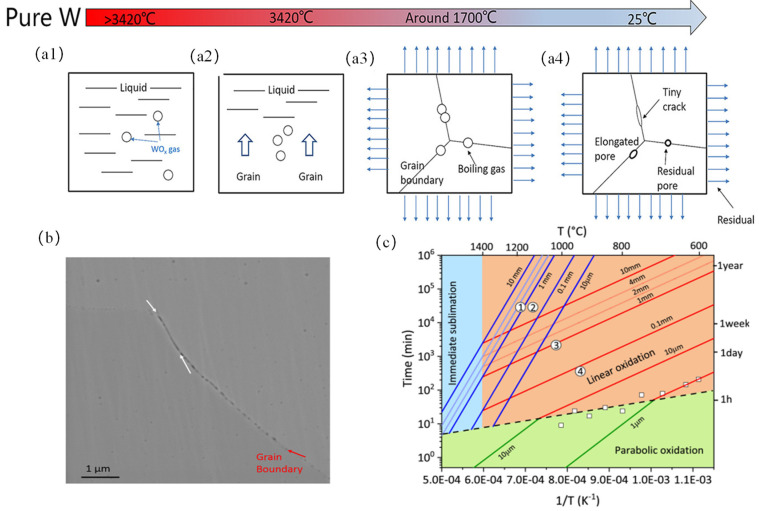
Schematic of pure W nanostructure formation in the laser melt pool: (**a1**) liquid state in the laser melt pool; (**a2**) W matrix begins to solidify; (**a3**) gas captured in the W matrix during boiling; (**a4**) nanoscale pores formed at room temperature [75]. (**b**) SEM images showing nanoscale pores in pure W [75]. (**c**) Oxidation mechanism diagram and quantified kinetics of pure W in the temperature range of 600–1600 °C [114].

As shown in Figure 6a (1–4), molten W is highly sensitive to oxygen and easily reacts with oxygen to form oxide inclusions such as WO_3_. When the tungsten liquid begins to solidify, WO_x_ remains in a gaseous state and is gradually squeezed into grain boundaries, eventually staying at the grain boundaries, forming pores and reducing the strength of the grain boundaries. As the temperature decreases, the W matrix will bear significant residual stress. When the residual stress exceeds the grain boundary strength, microcracks will appear [75]. Meanwhile, these inclusions form heterogeneous nucleation sites in the W matrix, leading to the weakening of grain boundaries, reducing their migration ability, and easily causing crack growth. The oxygen precipitated at high-angle grain boundaries also affects the DBTT transition in W materials [113].

Nagy et al. [114] conducted a more fundamental study on the oxidation process of W, proposing an oxidation mechanism diagram for tungsten. As shown in Figure 6c, it depicts the isopleths of metal recession thickness in the time temperature space for parabolic oxidation, linear oxidation, and sublimation kinetic growth laws. It was found that the transition from parabolic (protective) kinetics to linear (non-protective) kinetics is repeatable. The transition time decreases logarithmically with increasing temperature. Above approximately 1000 °C, the sublimation process becomes dominant, with a rate comparable to the oxidation rate in air at 1400 °C. Under atmospheric conditions. The activation energies for these three states are 118, 204 and 402 kJ/mol. The rate-limiting processes can be attributed to: (1) diffusion of oxygen vacancies in WO_3_; (2) surface reaction of 1.5O_2_ + W → WO_3_; and (3) evaporation of WO_3_.

As we have detailed the adverse effects of oxygen content on additive manufacturing of W materials, researchers have used radio frequency induction plasma to remove oxides from the tungsten powder surface to reduce oxygen content [115]. Wang et al. [75] prepared a W-6wt%Ta alloy by adding Ta to reduce the impact of oxygen content. The study showed that nanometer pores in W–6wt%Ta are mainly concentrated on the cell walls of the grains, and the gas pores between the grains are captured by the cell walls, resulting in fewer oxide pores at the grain boundaries in the W-Ta alloy. The strength GBs is significantly enhanced after Ta alloying, and microcracks are significantly fewer than in pure W alloy.

In the process of additive manufacturing W, the oxygen content will have an obvious adverse effect on the manufactured W materials, in the subsequent research, it can reduce the oxygen content in the construction chamber and reduce the oxygen content on the surface of the tungsten powder through pretreatment, which can effectively reduce the adverse effect of oxygen on the performance of the material. At the same time, the addition of secondary phase materials can also effectively reduce the cracks produced by oxygen in the manufacturing process, and thus the performance is effectively improved.

## 4. Additive Manufacturing of Tungsten Alloys

However, in some studies, altering the process parameters did not entirely produce defect-free W components. Therefore, researchers aimed to refine and alter the grain structure of AM-processed tungsten materials, inhibit cracks in W, enhance the inherent ductility of W, and improve the densification of tungsten parts. The exploration has included incorporating rare earth or other elements into pure tungsten to enhance its material properties. Tungsten alloying includes solid solution strengthened alloys and particle dispersion strengthened alloys. This section will discuss in detail the specific effects of various elements alloyed with tungsten on their mechanical properties.

Research shows that solid solution strengthened alloys are formed by alloying with Group IV or V transition metals (such as Ta, Ni, etc.) to create a solid solution forming new phases. These precipitates can limit the grain growth of W, alter the grain structure during solidification, and thereby increase the mechanical properties of tungsten. Additionally, the addition of alloying elements causes lattice distortion in tungsten, generating strain fields that hinder dislocation slip movements, thus enhancing the yield strength and hardness of W materials. Ivekovic et al. [56] mixed pure tungsten powder with 5 wt% and 10 wt% Ta powder, using internally developed SLM machines and 3D Systems industrial machines to fabricate W and W-Ta samples. In the case of pure tungsten, a checkerboard pattern without significant texture was observed, which became more pronounced with the addition of Ta. The addition of Ta resulted in a reduction in grain size. In samples fabricated using internally developed SLM machines, the maximum density of pure W was 94.4%TD, W-5wt%Ta was 94.1%TD, and W-10wt%Ta was 97.5%TD. The relative density of the samples increased with the Ta content. In contrast, samples fabricated using 3D Systems industrial machines had maximum densities of 97.1%TD for pure W, 98.7%TD for W-5wt%Ta, and 98.4%TD for W-10wt%Ta. This difference is attributed to the higher oxygen content in the chamber of the internal equipment (200 ppm, compared to 50 ppm in the 3D Systems equipment). The presence of oxygen and other impurities segregated at grain boundaries increased BDTT, thereby reducing surface energy and promoting intergranular fracture, which affected densification.

**Figure 7:**
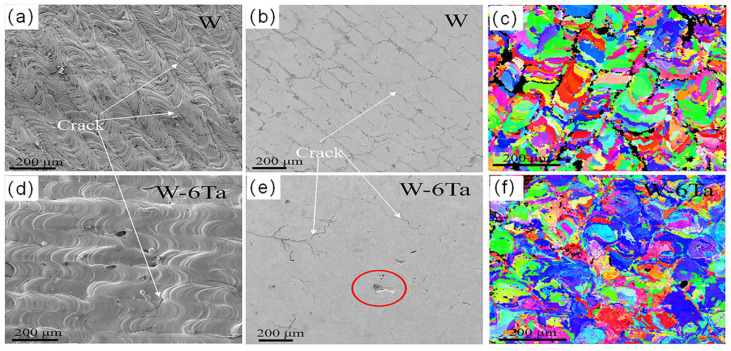
(**a**,**d**) the raw surface morphology, (**b**,**e**) the crack distribution, and the (**c**,**f**) EBSD of pure W and W-6Ta [75].

Another group, Wang et al. [75], reported the same method, as shown in Figure 7, finding that the cracks in W-6Ta bulk were reduced by 80% compared to pure W, with a crack density reduced to 2.0 mm^−1^. The cracks were shorter and more tortuous. Additionally, a honeycomb structure was observed in W–6Ta, with its cell walls composed of numerous dislocations, which could increase toughness. They found that the dislocation density in W-6Ta was higher than in W, supporting the proposal by Qi and Chrzan [116] that alloying promotes dislocation nucleation. After adding Ta, the strength increased by 13% compared to pure tungsten. The cellular structure within W-6Ta captures nanopores inside the grains, reducing oxide gas pores at the GBs, thereby increasing strength GBs.

**Figure 8:**
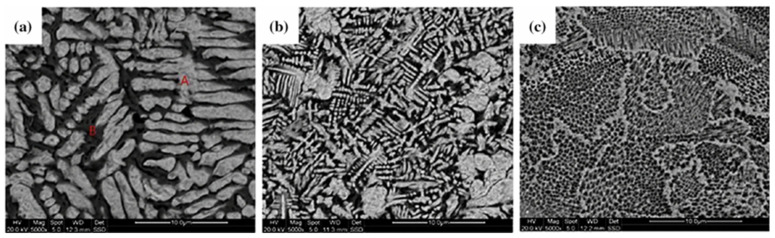
Microstructure of SLM-fabricated W–Ni samples: (**a**) Ni content of 10%; (**b**) Ni content of 20%; (**c**) Ni content of 40% [55].

Zhang et al. [55] studied the structural characteristics of W-Ni alloys with different Ni contents (10%, 20%, and 40%) using the L-PBF manufacturing process. The results showed that since Ni has a much lower melting temperature than W, the increase in Ni helps to increase the liquid content and reduce the temperature gradient. Under the same forming parameters, SLM samples with different Ni contents exhibited different microstructures. With the increase in Ni content, the microstructure of the samples evolved from strip structures to dendritic structures and then to honeycomb structures, with higher densification, as shown in Figure 8. Additionally, due to the shrinkage of the loose powder bed, the hardness of the W-Ni alloys formed with increased Ni content decreased along the building direction.

Yan and Wang et al. [58,117] used L-PBF to prepare W-(Ni)-Cu composites and explored the densification mechanism transition of W-(Ni)-Cu composites with different compositions. The results showed that with the increase of Cu content, the degree of densification increased, while the hardness decreased. The addition of Ni significantly enhanced the bonding between Cu and W, indicating that the addition of Ni facilitates the densification of the Cu matrix. The Ni-Cu phase tends to decompose the liquid W into small grains, forming nanocrystals, which result in grain refinement and, consequently, enhance the microhardness of the W phase. Bose et al. [118] found that the addition of alloy elements such as Mo, Ta, Cr, and Re results in finer grain structures. In this study, the highest hardness of W-based materials was reported. The W-Cr parts manufactured achieved a maximum hardness of 966 HV after sintering at 1500 °C for 1 h, with relative density comparable to traditional tungsten heavy alloys (WHA). Verdonik TW et al. [119] fabricated a 193W-5.6Ni-1.4Fe alloy with a density greater than 99.8% using the L-PBF process. Their study indicated that lower laser power combined with an extended exposure time can result in effective melting of the matrix phase, which helps to reduce the occurrence of microcracks.

**Figure 9:**
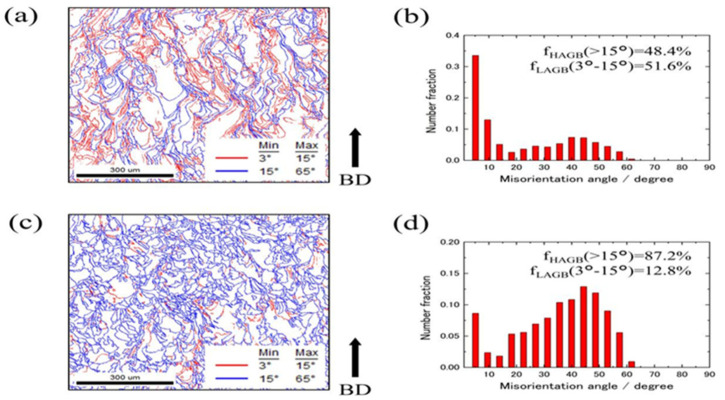
(**a**,**c**) show the grain boundary lines corresponding to the IPF maps, with red and blue lines representing LAGB and HAGB, respectively. (**b**,**d**) are histograms of the misorientation angle data [120].

**Figure 10:**
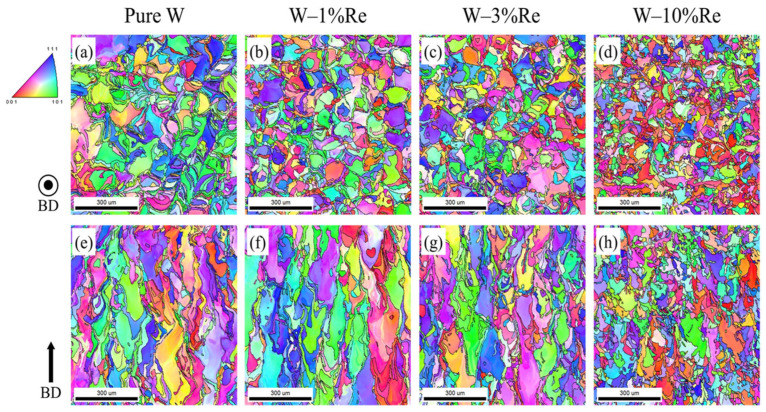
IPF maps of pure W and W-Re alloys. (**a**–**d**) are horizontal cross-sections; (**e**–**h**) are vertical cross-sections [120].

Xue et al. [57] found that Nb can suppress microcracks in SLMed W-Nb alloys. This is because the alloying element can compensate for the shrinkage stress as W solidifies first due to its high melting point, so alloying with Nb can provide solid solution strengthening and improve intergranular bonding. As shown in Figure 9a,e, the columnar grains in pure W are significantly elongated along the BD direction. Figure 10b–d,f–h show that with the increase of Re content, the inhibition of grain boundary migration leads to gradual grain refinement, and the average grain diameter gradually decreases. The average grain diameters of pure W on the horizontal and vertical sections are 57 ± 40 μm and 92 ± 60 μm, respectively, while the values for W-10%Re are 36 ± 19 μm and 48 ± 31 μm. The study also found that the addition of Re can significantly reduce the thermal diffusivity, thereby reducing the cooling rate of the solidification layer and leading to a lower temperature gradient at the melt pool boundary. This effectively reduces the cracking sensitivity of W alloys in the AM process. From the EBSD data in Figure 10., it is shown that the proportion of LAGBs and HAGBs in the grain boundary structure of L-PBF fabricated pure W is 51.6% and 48.4%. The grain boundary structure of W-10%Re is mainly composed of HAGBs. Although it has been proven that cracks are more likely to grow at HAGBs than at LAGBs in pure W, and the high proportion of HAGBs in W-10%Re may lead to increased oxygen segregation sites, the scattering effect of Re atoms on phonons and electrons increases the thermal resistance isotropy of the W matrix when a certain proportion of Re is added, reducing crack formation and effectively mitigating thermal anisotropy [120].

In general, the alloy phase formed by alloying can act as a binder during the laser additive manufacturing process to increase the densities of the prepared tungsten parts, and the alloying can form precipitates that limit the growth of W grains, thus refining the grain structure of the tungsten material and showing unique performance advantages.

## 5. Additive Manufacturing of Tungsten-Based Composites

Although solid solution strengthened tungsten alloys can improve the densification of tungsten parts, enhance grain structure, and reduce cracks, they have a lower melting temperature compared to pure tungsten and lose most of their strength at half the solidus temperature [121]. In contrast, particle dispersed strengthened tungsten alloys can maintain their strength even at high temperatures [1]. Particle dispersion strengthening involves introducing nano- or micron-sized second-phase particles (typically hard phases like carbides or oxides) into the tungsten matrix, forming obstacles that directly impede dislocation slip and climb, thereby enhancing the material properties of tungsten parts [122,123]. For example, ref. [124] found that peritectic reactions are possible during the non-equilibrium solidification between Fe and W. This provides more metallic phases, such as Fe_7_W_6_ and Fe_2_W, in W-Fe alloys, greatly increasing the material’s hardness. Furthermore, in the L-PBF process, planar epitaxial solidification often forms large columnar grains, leading to crack formation, while nanoparticles can provide heterogeneous nucleation sites before solidification, resulting in fine equiaxed grains and reducing semi-solid residual stress [125].

**Figure 11:**
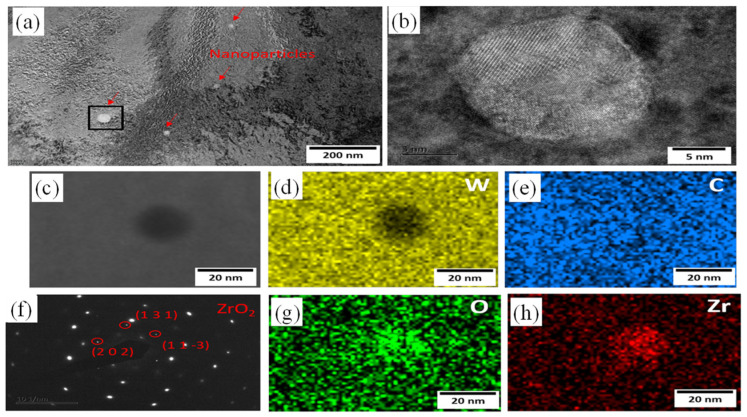
Analysis of the nanoparticles. (**a**) is bright field image showing the distribution of the nanoparticles, (**b**) is the HRTEM of the selected nanoparticle, (**c**) is the high-angle annular dark field (HAADF) image. (**d**,**e**,**g**,**h**) are the EDS maps of the selected nanoparticle at a different magnification and (**f**) is the SAEDP of the selected nanoparticle [126].

Li et al. [126] introduced secondary-phase ZrC nanoparticles into the W matrix to form W-ZrC composites, reducing the crack density by 88.7% compared to pure tungsten and significantly decreasing the average grain size in W-ZrC (5.6 μm) compared to pure W (10.1 μm). The fine-grained structure in W-ZrC has a larger grain boundary length-to-surface area ratio, leading to uniform residual stress distribution and increased crack propagation resistance. Additionally, ZrC captures oxygen impurities to form ZrO_2_ (as shown in Figure 11), reducing the embrittlement effect of oxygen impurities on grain boundaries.

Hu et al. [127] compared the effects of micron and nano Y_2_O_3_ particles, and the results showed that adding Y_2_O_3_ particles helps dispersion strengthening, thereby increasing microhardness. Due to the strong Marangoni convection effect caused by concentration differences, Y_2_O_3_ agglomerates in the um–ODS–W samples, but not in the nm-ODS-W samples. Moreover, the nano particles form many low-angle distorted tungsten grains in the W matrix, enhancing crack propagation resistance. For TaC particles, Ta alloying will change the Fermi level, giving W materials greater intrinsic ductility and helping reduce crack sensitivity. Chen et al. [128] added 5 wt% TaC into the W matrix. According to the W-Ta thermodynamic phase diagram, W and Ta can form an infinite solid solution, and through the route of Equation (16), an in-situ reaction forms a secondary phase W_2_C. A similar approach to [126] was proposed, where the formation of TaO_x_ and C removes oxygen and powder from the chamber, purifying the grain boundaries and thereby inhibiting crack initiation and propagation.

(16) $$W+\mathrm{TaC}\;\to \;W\text{-}\mathrm{Ta}\text{-}C\left(\mathrm{liquid}\right)\to \;W\left(\mathrm{Ta}\right)+W_{2}C$$

Stackhouse et al. [129] reported that for pure tungsten samples, the number of transverse cracks decreases with increased power and is independent of scan speed, whereas for W-CeO_2_, W-La_2_O_3_, and W-Rare alloy samples, the number of cracks linearly increases with increasing scan speed. In the L-PBF process, by selecting appropriate process parameters, W-based composites can reduce crack formation in AMed parts through grain refinement and the introduction of more low-angle grain boundaries (LAGB) to reduce grain boundary residual stress, thereby improving material hardness and achieving superior material properties [125,130]. In another study, Gu et al. [131] used 2.5 wt% TiC nanoparticles and W particles, achieving complete melting of all elements and forming crack-free W-Ti-W alloy samples, with the processed parting density reaching near-full densification at 97.8%.

The above part answers well the mechanism of carbides and oxides behaving differently in W-matrix composites and provides a good design guide for L-PBF fabricated W materials. In the subsequent study, the reasonably designed W-matrix composites can be designed by grain refinement and trapping of oxygen impurities in the grain boundaries in order to obtain excellent mechanical properties. Meanwhile, more secondary phase particles are experimented to further investigate whether and how they can regulate DBTT and solve the cracking problem of AMed W materials.

## 6. Conclusions

Tungsten is a refractory metal with a wide range of applications, becoming an indispensable material in aerospace, nuclear industries, and high-temperature engineering. With the continuous development of additive manufacturing (AM) technology, combining it with tungsten can overcome the drawbacks of traditional tungsten manufacturing, better adapting to the complex structural demands of various scenarios. L-PBF brings higher system design flexibility and more outstanding performance to the fabrication of tungsten materials. In order in better research on L-PBF fabricated tungsten materials, this review provides important theoretical know-how on the challenging problems in L-PBF fabricated W materials.

Currently, many researchers are experimenting with the manufacturability of W-based materials using L-PBF technology. In this article, we review the multi-scale thermodynamic behavior of L-PBF manufacturing of tungsten, conducting quantitative analysis on melt pool dynamics, crystal plasticity analysis, and grain growth mechanisms. By summarizing their governing equations, the fun cracking in laser additively manufactured W, Initiation mechanism, and a suppression approach by alloying fundamental principles of L-PBF manufacturing of tungsten can be well understood.

Despite the high melting temperature and high ductile-to-brittle transition temperature (DBTT) of tungsten and its alloys making it difficult to manufacture defect-free parts, advancements in AM processes over the past decade have shown the potential for producing perfect tungsten components. Although cracking and densification remain significant challenges, improvements in L-PBF manufacturing processes can directly enhance the densification and crack resistance of AMed tungsten materials. Different processing parameters significantly affect the microstructure and performance of L-PBF manufactured tungsten materials. This paper comprehensively analyzes the impact of parameters such as volumetric energy density, scanning strategy, and substrate preheating on tungsten processing and provides the optimal processing parameters. Additionally, oxygen content levels are crucial in the L-PBF manufacturing of tungsten. Removing oxides from the tungsten powder surface using radio frequency induction plasma, reducing the oxygen content in the building chamber, and introducing Ta elements can effectively mitigate the impact of oxygen during the melting process on tungsten materials.

To improve the performance of AMed W materials, different alloying elements (e.g., Ta, Ni, Cu, Nb, Mn, Re, Cr) have been introduced into W materials, and their effects have been extensively studied and summarized. The impact on microstructure, mechanical properties, microcracks, and densification has been systematically clarified. Additionally, the mechanisms of secondary phase particles (e.g., ZrC, La_2_O_3_, Y_2_O_3_, TiC) with the W matrix have been thoroughly explained, effectively reducing microcracks during manufacturing and adjusting DBTT. Further exploration of new alloy designs and composite material technologies in the future will aid in developing more adaptable tungsten-based materials.

In summary, L-PBF technology offers new possibilities for the development of tungsten, tungsten alloys, and tungsten-based composites, with the application range of tungsten materials expected to expand further in the future. This review provides a solid foundation for understanding and developing high-quality, high-performance AMed W materials.

## Figures and Tables

**Table 1:** Summary of the effect of different oxygen contents on performance.

Machine	Powder	Oxygen Content	Laser Power (W)	Energy Density (J/mm^3^)	Max Density (%)	Cracking	Ref.
KU Leuven	Pure W	150–200 ppm	-	200–900	94.40%	Yes	[56]
3DSystems ProX^®^ DMP 320		<50 ppm	-		97.10%	Yes. Less cracks	
Renishaw AM400 machine	Pure W	<100 ppm	400 W	-	96.00%	Yes	[75]
	W-6wt%			-	96.50%	Yes. Cracks reduced by 80%	
Lab–scale SLM machine equipped	Pure W	800 ppm	400 W	-	97.50%	Yes	[113]
		20 ppm		-	97.50%	Yes. No continuous crack network	

## Data Availability

The raw data supporting the conclusions of this article will be made available by the authors on request.

## References

[1] Lassner E., Schubert W.D. (1999). Tungsten: Properties, Chemistry, Technology of the Element, Alloys, and Chemical Compounds.

[2] Norajitra P., Boccaccini L., Diegele E., Filatov V., Gervash A., Giniyatulin R., Gordeev S., Heinzel V., Janeschitz G., Konys J. (2004). Development of a helium-cooled divertor concept: Design-related requirements on materials and fabrication technology. J. Nucl. Mater..

[3] Katoh Y., Snead L., Garrison L., Hu X., Koyanagi T., Parish C., Edmondson P., Fukuda M., Hwang T., Tanaka T. (2019). Response of unalloyed tungsten to mixed spectrum neutrons. J. Nucl. Mater..

[4] Wei Q., Ramesh K.T., Schuster B.E., Kecskes L.J., Dowding R.J. (2006). Nanoengineering opens a new era for tungsten as well. JOM.

[5] Zinkle S.J., Ott L.J., Ingersoll D.T., Ellis R.J., Grossbeck M.L. (2002). Overview of materials technologies for space nuclear power and propulsion. AIP Conf. Proc..

[6] Wurster S., Baluc N., Battabyal M., Crosby T., Du J., García-Rosales C., Hasegawa A., Hoffmann A., Kimura A., Kurishita H. (2013). Recent progress in R&D on tungsten alloys for divertor structural and plasma facing materials. J. Nucl. Mater..

[7] Oponowicz A., Marciszko-Wiąckowska M., Baczmański A., Klaus M., Genzel C., Wroński S., Kollbek K., Wróbel M. (2020). Gradient of Residual Stress and Lattice Parameter in Mechanically Polished Tungsten Measured Using Classical X-rays and Synchrotron Radiation. Met. Mater. Trans. A.

[8] Gear J.I., Taprogge J., White O., Flux G.D. (2019). Characterisation of the attenuation properties of 3D-printed tungsten for use in gamma camera collimation. EJNMMI Phys..

[9] Sidambe A., Judson D., Colosimo S., Fox P. (2019). Laser powder bed fusion of a pure tungsten ultra-fine single pinhole collimator for use in gamma ray detector characterisation. Int. J. Refract. Met. Hard Mater..

[10] Pan S.H., Yao G.C., Cui Y.N., Meng F.S., Luo C., Zheng T.Q., Singh G. (2023). Additive manufacturing of tungsten, tungsten-based alloys, and tungsten matrix composites. Tungsten.

[11] Wei C., Liu L., Gu Y., Huang Y., Chen Q., Li Z., Li L. (2022). Multi-material additive-manufacturing of tungsten—Copper alloy bimetallic structure with a stainless-steel interlayer and associated bonding mechanisms. Addit. Manuf..

[12] Ellis E.A., Sprayberry M.A., Ledford C., Hankwitz J.P., Kirka M.M., Rock C.D., Horn T.J., Katoh Y., Dehoff R.R. (2021). Processing of tungsten through electron beam melting. J. Nucl. Mater..

[13] Seeger A. (2002). Peierls barriers, kinks, and flow stress: Recent progress. Int. J. Mater. Res..

[14] Gumbsch P., Riedle J., Hartmaier A., Fischmeister H.F. (1998). Controlling Factors for the Brittle-to-Ductile Transition in Tungsten Single Crystals. Science.

[15] Bonnekoh C., Hoffmann A., Reiser J. (2018). The brittle-to-ductile transition in cold rolled tungsten: On the decrease of the brittle-to-ductile transition by 600K to 65°C—ScienceDirect. Int. J. Refract. Met. Hard Mater..

[16] Zhou X., Liu X., Zhang D., Shen Z., Liu W. (2015). Balling phenomena in selective laser melted tungsten. J. Mater. Process. Technol..

[17] Liu R., Xie Z., Hao T., Zhou Y., Wang X., Fang Q., Liu C. (2014). Fabricating high performance tungsten alloys through zirconium micro-alloying and nano-sized yttria dispersion strengthening. J. Nucl. Mater..

[18] Farid N., Zhao D., Oderji H., Ding H. (2014). Cracking and damage behavior of tungsten under ELM’s like energy loads using millisecond laser pulses. J. Nucl. Mater..

[19] Ma J., Zhang J., Liu W., Shen Z. (2013). Suppressing pore-boundary separation during spark plasma sintering of tungsten. J. Nucl. Mater..

[20] Neu R., Coenen J.W., Curzadd B., Gietl H., Greuner H., Höschen T., You J.H. (2023). Material and component developments for the DEMO divertor using fibre reinforcement and additive manu-facturing. Mater. Res. Express.

[21] Li K.-L., Chen J.-H., Zhao C.-C., Shen Z.-J., Liu W. (2021). A review of tungsten fabricated via laser powder bed fusion. Tungsten.

[22] Omole S., Lunt A., Kirk S., Shokrani A. (2022). Advanced processing and machining of tungsten and its alloys. J. Manuf. Mater. Process.

[23] Guo M., Gu D., Xi L., Du L., Zhang H., Zhang J. (2019). Formation of scanning tracks during Selective Laser Melting (SLM) of pure tungsten powder: Morphology, geometric features and forming mechanisms. Int. J. Refract. Met. Hard Mater..

[24] Alfaify A., Saleh M., Abdullah F., Al-Ahmari A. (2020). Design for Additive Manufacturing: A Systematic Review. Sustainability.

[25] Sefene E.M. (2022). State-of-the-art of selective laser melting process: A comprehensive review. J. Manuf. Syst..

[26] Sefene E.M., Hailu Y.M., Tsegaw A.A. (2022). Metal hybrid additive manufacturing: State-of-the-art. Prog. Addit. Manuf..

[27] Fereiduni E., Ghasemi A., Elbestawi M. (2020). Selective Laser Melting of Aluminum and Titanium Matrix Composites: Recent Progress and Potential Applications in the Aerospace Industry. Aerospace.

[28] Song Y., Yan Y., Zhang R., Xu D., Wang F. (2002). Manufacture of the die of an automobile deck part based on rapid prototyping and rapid tooling technology—ScienceDirect. J. Mater. Process. Technol..

[29] Zhang L., Attar H. (2015). Selective Laser Melting of Titanium Alloys and Titanium Matrix Composites for Biomedical Applications: A Review. Adv. Eng. Mater..

[30] Kutzer M.D., DeVries L.D. (2017). Testbed for Multilayer Conformal Additive Manufacturing. Technologies.

[31] Deprez K., Vandenberghe S., Van Audenhaege K., Van Vaerenbergh J., Van Holen R. (2012). Rapid additive manufacturing of MR compatible multipinhole collimators with selective laser melting of tungsten powder. Med. Phys..

[32] Thijs L., Sistiaga M.L.M., Wauthle R., Xie Q., Kruth J.-P., Van Humbeeck J. (2013). Strong morphological and crystallographic texture and resulting yield strength anisotropy in selective laser melted tantalum. Acta Mater..

[33] Faidel D., Jonas D., Natour G., Behr W. (2015). Investigation of the selective laser melting process with molybdenum powder. Addit. Manuf..

[34] Dong Z., Han C., Zhao Y., Huang J., Ling C., Hu G., Wang Y., Wang D., Song C., Yang Y. (2024). Role of heterogenous microstructure and deformation behavior in achieving superior strength-ductility synergy in zinc fabricated via laser powder bed fusion. Int. J. Extrem. Manuf..

[35] Neuberger H., Hernandez F., Rey J., Bonk S., Rieth M., Koch J., Schmalisch B., Müller O., Volker K.-U., Volker D. (2020). Fabrication of HCPB breeding blanket components using the additive manufacturing processes of selective laser melting and cold spray. Fusion. Eng. Des..

[36] Zhou S., Su Y., Wang H., Enz J., Ebel T., Yan M. (2020). Selective laser melting additive manufacturing of 7xxx series Al-Zn-Mg-Cu alloy: Cracking elimination by co-incorporation of Si and TiB2. Addit. Manuf..

[37] Biffi C.A., Bassani P., Fiocchi J., Albu M., Tuissi A. (2021). Selective laser melting of AlCu-TiB 2 alloy using pulsed wave laser emission mode: Processability, microstructure and mechanical properties. Mater. Des..

[38] Yadroitsev I., Krakhmalev P., Yadroitsava I. (2014). Selective laser melting of Ti6Al4V alloy for biomedical applications: Temperature monitoring and mi-crostructural evolution. J. Alloys Compd..

[39] Baudana G., Biamino S., Ugues D., Lombardi M., Fino P., Pavese M., Badini C. (2016). Titanium aluminides for aerospace and automotive applications processed by Electron Beam Melting: Contribution of Politecnico di Torino. Met. Powder Rep..

[40] Kirka M.M., Unocic K.A., Raghavan N., Medina F., Dehoff R.R., Babu S.S. (2016). Microstructure Development in Electron Beam-Melted Inconel 718 and Associated Tensile Properties. JOM.

[41] Gu D.D., Meiners W., Wissenbach K., Poprawe R. (2012). Laser additive manufacturing of metallic components: Materials, processes and mechanisms. Int. Mater. Rev..

[42] Thompson S.M., Bian L., Shamsaei N., Yadollahi A. (2015). An overview of Direct Laser Deposition for additive manufacturing; Part I: Transport phenomena, modeling and diagnostics. Addit. Manuf..

[43] Müller A.V., Schlick G., Neu R., Anstätt C., Klimkait T., Lee J., Pascher B., Schmitt M., Seidel C. (2019). Additive manufacturing of pure tungsten by means of selective laser beam melting with substrate preheating temperatures up to 1000 °C. Nucl. Mater. Energy.

[44] Patterson A.E., Messimer S.L., Farrington P.A. (2019). Overhanging Features and the SLM/DMLS Residual Stresses Problem: Review and Future Research Need. Technologies.

[45] Niu P., Li R., Gan K., Fan Z., Yuan T., Han C. (2024). Manipulating Stacking Fault Energy to Achieve Crack Inhibition and Superior Strength–Ductility Synergy in an Additively Manufactured High-Entropy Alloy. Adv. Mater..

[46] Wu Y., Sun K., Yu S., Zuo L. (2020). Modeling the selective laser melting-based additive manufacturing of thermoelectric powders. Addit. Manuf..

[47] Yan W., Lin S., Kafka O.L., Lian Y., Yu C., Liu Z., Liu W.K. (2018). Data-driven multi-scale multi-physics models to derive process–structure–property relationships for ad-ditive manufacturing. Comput. Mech..

[48] Rosenthal D. (1941). Mathematical Theory of Heat Distribution during Welding and Cutting. Weld. J..

[49] Todo T., Ishimoto T., Gokcekaya O., Oh J., Nakano T. (2021). Single crystalline-like crystallographic texture formation of pure tungsten through laser powder bed fusion. Scr. Mater..

[50] Wang C., Li Z.J., Ji C.Q., Gao S.W., Cui Y.N. (2022). Crystal plasticity analysis of the evolutions of temperature, stress and dislocation in additively manufactured tungsten. Int. J. Refract. Met. Hard Mater..

[51] Goldak J., Chakravarti A., Bibby M. (1984). A new finite element model for welding heat sources. Metall. Trans. B.

[52] Tan C., Zhou K., Ma W., Attard B., Zhang P., Kuang T. (2018). Selective laser melting of high-performance pure tungsten: Parameter design, densification behavior and mechanical properties. Sci. Technol. Adv. Mater..

[53] Ng GK L., Jarfors AE W., Bi G., Zheng H.Y. (2009). Porosity formation and gas bubble retention in laser metal deposition. Appl. Phys. A.

[54] Wu W.H., Yang Y.Q., Wang D. (2010). Balling Phenomenon in Selective Laser Melting Process. J. S. China Univ. Technol..

[55] Zhang D., Liu Z., Cai Q., Liu J., Chua C. (2014). Influence of Ni content on microstructure of W–Ni alloy produced by selective laser melting. Int. J. Refract. Met. Hard Mater..

[56] Iveković A., Omidvari N., Vrancken B., Lietaert K., Thijs L., Vanmeensel K., Kruth J.P. (2018). Selective laser melting of tungsten and tungsten alloys. Int. J. Refract. Met. Hard Mater..

[57] Xue J., Feng Z., Tang J., Tang C., Zhao Z. (2021). Selective laser melting additive manufacturing of tungsten with niobium alloying: Micro-structure and suppression mechanism of microcracks. J. Alloys Compd. Interdiscip. J. Mater. Sci. Solid-State Chem. Phys..

[58] Yan A., Wang Z., Yang T., Wang Y., Ma Z. (2016). Microstructure, thermal physical property and surface morphology of W-Cu compositefabricated via selective laser melting. Mater. Des..

[59] (2001). Meggyes, Multiple decomposition in finite deformation theory. Acta Mech..

[60] McAuliffe C., Waisman H. (2015). A unified model for metal failure capturing shear banding and fracture. Int. J. Plast..

[61] Roters F., Eisenlohr P., Hantcherli L., Tjahjanto D., Bieler T., Raabe D. (2009). Overview of constitutive laws, kinematics, homogenization and multiscale methods in crystal plasticity finite-element modeling: Theory, experiments, applications. Acta Mater..

[62] Bayat M., Dong W., Thorborg J., To A.C., Hattel J.H. (2021). A review of multi-scale and multi-physics simulations of metal additive manufacturing processes with focus on modeling strategies. Addit. Manuf..

[63] Cao J., Gharghouri M.A., Nash P. (2016). Finite-element analysis and experimental validation of thermal residual stress and distortion in electron beam additive manufactured Ti-6Al-4V build plates. J. Am. Acad. Dermatol..

[64] Hirsch P.B. (1957). Elements of X-Ray Diffraction. Phys. Bull..

[65] De Baere D., Van Cauwenbergh P., Bayat M., Mohanty S., Thorborg J., Thijs L., Van Hooreweder B., Vanmeensel K., Hattel J.H. (2021). Thermo-mechanical modelling of stress relief heat treatments after laser-based powder bed fusion. Addit. Manuf..

[66] Du Z.-Y., Lv Y.-Q., Han Y., Fan J.-L., Ye L. (2020). Sintering densification behavior and kinetic mechanism of nano-tungsten powder prepared by sol-spray drying. Tungsten.

[67] Thompson F.C. (1944). Principles of Powder Metallurgy. Nature.

[68] Wang H., Fang Z.Z., Hwang K.S. (2011). Kinetics of Initial Coarsening During Sintering of Nanosized Powders. Met. Mater. Trans. A.

[69] Huang J., Li W., Yang T., Chou T.-H., Zhou R., Liu B., Huang J.C., Liu Y. (2024). An additively manufactured precipitation hardening medium entropy alloy with excellent strength-ductility synergy over a wide temperature range. J. Mater. Sci. Technol..

[70] Zhang D., Cai Q., Liu J. (2012). Formation of Nanocrystalline Tungsten by Selective Laser Melting of Tungsten Powder. Mater. Manuf. Process..

[71] Enneti R.K., Morgan R., Atre S.V. (2017). Effect of process parameters on the Selective Laser Melting (SLM) of tungsten. Int. J. Refract. Met. Hard Mater..

[72] Wang D.-Z., Li K.-L., Yu C.-F., Ma J., Liu W., Shen Z.-J. (2018). Cracking Behavior in Additively Manufactured Pure Tungsten. Acta Met. Sin..

[73] Zhou K., Chen W., Yang Y., Li R., Dong L., Fu Y.-Q. (2022). Microstructure and mechanical behavior of porous tungsten skeletons synthesized by selected laser melting. Int. J. Refract. Met. Hard Mater..

[74] Vrancken B., Ganeriwala R.K., Matthews M.J. (2020). Analysis of laser-induced microcracking in tungsten under additive manufacturing conditions: Experiment and simulation. Acta Mater..

[75] Wang D., Wang Z., Li K., Ma J., Liu W., Shen Z. (2018). Cracking in laser additively manufactured W: Initiation mechanism and a suppression approach by alloying. Mater. Des..

[76] Xu J., Lin X., Guo P., Hu Y., Wen X., Xue L., Liu J., Huang W. (2017). The effect of preheating on microstructure and mechanical properties of laser solid forming IN-738LC alloy. Mater. Sci. Eng. A.

[77] Liu Y.J., Li S.J., Wang H.L., Hou W.T., Hao Y.L., Yang R., Zhang L.C. (2016). Microstructure, defects and mechanical behavior of beta-type titanium porous structures manufactured by electron beam melting and selective laser melting—ScienceDirect. Acta Mater..

[78] Sing S., Huang S., Goh G., Tey C., Tan J., Yeong W. (2021). Emerging metallic systems for additive manufacturing: In-situ alloying and multi-metal processing in laser powder bed fusion. Prog. Mater. Sci..

[79] Simonelli M., Tuck C., Aboulkhair N.T., Maskery I., Ashcroft I., Wildman R.D., Hague R. (2015). A Study on the Laser Spatter and the Oxidation Reactions During Selective Laser Melting of 316L Stainless Steel, Al-Si10-Mg, and Ti-6Al-4V. Metall. Mater. Trans. A.

[80] Kumar L.J., Pandey P.M., Wimpenny D.I. (2018). 3D Printing and Additive Manufacturing Technologies.

[81] Ciurana J., Hernandez L., Delgado J. (2013). Energy density analysis on single tracks formed by selective laser melting with CoCrMo powder material. Int. J. Adv. Manuf. Technol..

[82] Donik C., Kraner J., Paulin I., Godec M. (2020). Influence of the Energy Density for Selective Laser Melting on the Microstructure and Mechanical Properties of Stainless Steel. Metals.

[83] Gong H., Rafi K., Gu H., Ram G.D.J., Starr T., Stucker B. (2015). Influence of defects on mechanical properties of Ti–6Al–4V components produced by selective laser melting and electron beam melting. Mater. Des..

[84] Gong H., Rafi K., Gu H., Starr T., Stucker B. (2014). Analysis of defect generation in Ti–6Al–4V parts made using powder bed fusion additive manufacturing pro-cesses—ScienceDirect. Addit. Manuf..

[85] Li Y., Gu D. (2014). Parametric analysis of thermal behavior during selective laser melting additive manufacturing of aluminum alloy powder. Mater. Des..

[86] Yang J., Han J., Yu H., Yin J., Gao M., Wang Z., Zeng X. (2016). Role of molten pool mode on formability, microstructure and mechanical properties of selective laser melted Ti-6Al-4V alloy. Mater. Des..

[87] Chen H., Zi X., Han Y., Dong J., Liu S., Chen C. (2019). Microstructure and mechanical properties of additive manufactured W-Ni-Fe-Co composite produced by selective laser melting. Int. J. Refract. Met. Hard Mater..

[88] Yuan W., Chen H., Cheng T., Wei Q. (2020). Effects of laser scanning speeds on different states of the molten pool during selective laser melting: Simulation and experiment. Mater. Des..

[89] Shi Q., Du W., Qin F., Tan C., Khanlari K., Xie H., Liu X., Wu A. (2023). Pure Tungsten Fabricated by Laser Powder Bed Fusion with Subsequent Hot Isostatic Pressing: Microstructural Evolution, Mechanical Properties, and Thermal Conductivity. J. Mater. Eng. Perform..

[90] Mishra P., Ilar T., Brueckner F., Kaplan A. (2018). Energy efficiency contributions and losses during selective laser melting. J. Laser Appl..

[91] Yang Y., Wen S., Wei Q., Li W., Liu J., Shi Y. (2017). Effect of scan line spacing on texture, phase and nanohardness of TiAl/TiB 2 metal matrix composites fabricated by selective laser melting. J. Alloys Compd. Interdiscip. J. Mater. Sci. Solid-State Chem. Phys..

[92] Shao M., Vijayan S., Nandwana P., Jinschek J.R. (2020). The effect of beam scan strategies on microstructural variations in Ti-6Al-4V fabricated by electron beam powder bed fusion. Mater. Des..

[93] Mercelis P., Kruth J.P. (2006). Residual stresses in selective laser sintering and selective laser melting. Rapid Prototyp. J..

[94] Kruth J.P., Froyen L., Van Vaerenbergh J., Mercelis P., Rombouts M., Lauwers B. (2004). Selective laser melting of iron-based powder. J. Mater. Process. Tech..

[95] Robinson J.H., Ashton I.R.T., Jones E., Fox P., Sutcliffe C. (2019). The effect of hatch angle rotation on parts manufactured using selective laser melting. Rapid Prototyp. J..

[96] Sillars S.A., Sutcliffe C.J., Philo A.M., Brown S.G.R., Sienz J., Lavery N.P. (2017). The three-prong method: A novel assessment of residual stress in laser powder bed fusion. Virtual Phys. Prototyp..

[97] Robinson J., Ashton I., Fox P., Jones E., Sutcliffe C. (2018). Determination of the effect of scan strategy on residual stress in laser powder bed fusion additive manufacturing. Addit. Manuf..

[98] Fang Z., Jing C., Lei X., Fengying Z., Xin L., Weidong H. (2010). Microstructure and Mechanical Properties of Laser Solid Formed Ti60 Alloy. Rare Met. Mater. Eng..

[99] Geiger F., Kunze K., Etter T. (2016). Tailoring the texture of IN738LC processed by selective laser melting (SLM) by specific scanning strategies. Mater. Sci. Eng. A.

[100] Nandwana P., Lee Y. (2020). Influence of scan strategy on porosity and microstructure of Ti-6Al-4V fabricated by electron beam powder bed fusion. Mater. Today Commun..

[101] Aboulkhair N.T., Maskery I., Tuck C., Ashcroft I., Everitt N.M. (2016). The microstructure and mechanical properties of selectively laser melted AlSi10Mg: The effect of a conventional T6-like heat treatment. Mater. Sci. Eng. A.

[102] Jia H., Sun H., Wang H., Wu Y., Wang H. (2021). Scanning strategy in selective laser melting (SLM): A review. Int. J. Adv. Manuf. Technol..

[103] Shipley H., McDonnell D., Culleton M., Coull R., Lupoi R., O’Donnell G., Trimble D. (2018). Optimisation of process parameters to address fundamental challenges during selective laser melting of Ti-6Al-4V: A review. Int. J. Mach. Tools Manuf..

[104] Sheng H., Wai Yee Y. Laser re-scanning strategy in selective laser melting for part quality enhancement: A review. Proceedings of the 3rd International Conference on Progress in Additive Manufacturing.

[105] Carter L.N., Martin C., Withers P.J., Attallah M.M. (2014). The influence of the laser scan strategy on grain structure and cracking behaviour in SLM powder-bed fabricated nickel superalloy. J. Alloys Compd. Interdiscip. J. Mater. Sci. Solid-State Chem. Phys..

[106] Wan H., Zhou Z., Li C., Chen G., Zhang G. (2019). Effect of scanning strategy on mechanical properties of selective laser melted Inconel 718. Mater. Sci. Eng. A.

[107] Thijs L., Verhaeghe F., Craeghs T., Van Humbeeck J., Kruth J.P. (2010). A study of the microstructural evolution during selective laser melting of Ti–6Al–4V. Acta Mater..

[108] Larimian T., Kannan M., Grzesiak D., AlMangour B., Borkar T. (2019). Effect of energy density and scanning strategy on densification, microstructure and mechanical properties of 316L stainless steel processed via selective laser melting. Mater. Sci. Eng. A.

[109] Liu C.Y., Tong J.D., Jiang M.G., Chen Z.W., Xu G., Liao H.B., Lao C.S. (2019). Effect of scanning strategy on microstructure mechanical properties of selective laser melted reduced activation ferritic/martensitic steel. Mater. Sci. Eng. A.

[110] Talignani A., Seede R., Whitt A., Zheng S., Ye J., Karaman I., Wang Y.M. (2022). A review on additive manufacturing of refractory tungsten and tungsten alloys. Addit. Manuf..

[111] Carpenter K., Tabei A. (2020). On Residual Stress Development, Prevention, and Compensation in Metal Additive Manufacturing. Ind. Organi-Zational Psychol..

[112] Vrancken B., Ganeriwala R.K., Martin A.A., Matthews M.J. (2021). Microcrack mitigation during laser scanning of tungsten via preheating and alloying strategies. Addit. Manuf..

[113] Braun J., Kaserer L., Stajkovic J., Leitz K.H., Tabernig B., Singer P., Leichtfried G. (2019). Molybdenum and tungsten manufactured by selective laser melting: Analysis of defect structure and solidification mechanisms. Int. J. Refract. Met. Hard Mater..

[114] Nagy D., Humphry-Baker S.A. (2021). An oxidation mechanism map for tungsten. Scr. Mater..

[115] Wang L.-Z., Wu J.-J., Zhang D.-J. (2017). Properties evolution of additive manufacture used tungsten powders prepared by radio frequency induction plasma. Int. J. Refract. Met. Hard Mater..

[116] Qi L., Chrzan D.C. (2014). Tuning Ideal Tensile Strengths and Intrinsic Ductility of bcc Refractory Alloys. Phys. Rev. Lett..

[117] Wang M., Li R., Yuan T., Chen C., Zhang M., Weng Q., Yuan J. (2018). Selective laser melting of W-Ni-Cu composite powder: Densification, microstructure evolution and nano-crystalline formation. Int. J. Refract. Met. Hard Mater..

[118] Bose A., Schuh C.A., Tobia J.C., Tuncer N., Mykulowycz N.M., Preston A., Lund A.C. (2018). Traditional and additive manufacturing of a new Tungsten heavy alloy alternative. Int. J. Refract. Met. Hard Mater..

[119] Verdonik T.W., Pires M., Guo T.-S., Rockwell N., Misiolek W.Z. (2023). Investigation of powder bed laser fusion additive manufacturing of 93W, 5.6Ni, 1.4Fe tungsten heavy alloys. Int. J. Refract. Met. Hard Mater..

[120] Yamamoto T., Hara M., Hatano Y. (2021). Cracking behavior and microstructural, mechanical and thermal charac-teristics of tungsten–rhenium binary alloys fabricated by laser powder bed fusion. Int. J. Refract. Met. Hard Mater..

[121] Deng H., Xie Z., Wang Y., Liu R., Zhang T., Hao T., Wang X., Fang Q., Liu C. (2018). Mechanical properties and thermal stability of pure W and W-0.5 wt%ZrC alloy manufactured with the same technology. Mater. Sci. Eng. A.

[122] Pan S., Jin K., Wang T., Zhang Z., Zheng L., Umehara N. (2022). Metal matrix nanocomposites in tribology: Manufacturing, performance, and mechanisms. Friction.

[123] Xie Z.M., Liu R., Miao S., Yang X.D., Zhang T., Wang X.P., Liu X. (2015). Extraordinary high ductility/strength of the interface designed bulk W-ZrC alloy plate at relatively low temperature. Sci. Rep..

[124] Chen H., Ye L., Han Y., Chen C., Fan J. (2021). Additive manufacturing of W-Fe composites using laser metal deposition: Microstructure, phase transfor-mation, and mechanical properties. Mater. Sci. Eng. A Struct. Mater. Prop. Misrostructure Process..

[125] Martin J.H., Yahata B.D., Hundley J.M., Mayer J.A., Schaedler T.A., Pollock T.M. (2017). 3D printing of high-strength aluminium alloys. Nature.

[126] Li K., Wang D., Xing L., Wang Y., Yu C., Chen J., Zhang T., Ma J., Liu W., Shen Z. (2018). Crack suppression in additively manufactured tungsten by introducing secondary-phase nanoparticles into the matrix. Int. J. Refract. Met. Hard Mater..

[127] Hu Z., Zhao Y., Guan K., Wang Z., Ma Z. (2020). Pure tungsten and oxide dispersion strengthened tungsten manufactured by selective laser melting: Mi-crostructure and cracking mechanism. Addit. Manuf..

[128] Chen J., Zhao C., Li K., Li M., Sun S., Zhang S., Ma J., Liu W. (2022). Effect of TaC addition on microstructure and microhardness of additively manufactured tungsten. J. Alloy. Compd..

[129] Stackhouse N.T. (2019). Tungsten Alloy Laser Track Cracking Analysis.

[130] Wu Y. (2019). Manufacturing of tungsten and tungsten composites for fusion application via different routes. Tungsten.

[131] Gu D., Dai D., Chen W., Chen H. (2016). SelectiveLaser Melting Additive Manufacturing of Hard-to-Process Tungsten-BasedAlloy Parts With Novel Crystalline Growth Morphology Enhanced Performance. J. Manuf. Sci. Eng..

